# PDBe: improved accessibility of macromolecular structure data from PDB and EMDB

**DOI:** 10.1093/nar/gkv1047

**Published:** 2015-10-17

**Authors:** Sameer Velankar, Glen van Ginkel, Younes Alhroub, Gary M. Battle, John M. Berrisford, Matthew J. Conroy, Jose M. Dana, Swanand P. Gore, Aleksandras Gutmanas, Pauline Haslam, Pieter M. S. Hendrickx, Ingvar Lagerstedt, Saqib Mir, Manuel A. Fernandez Montecelo, Abhik Mukhopadhyay, Thomas J. Oldfield, Ardan Patwardhan, Eduardo Sanz-García, Sanchayita Sen, Robert A. Slowley, Michael E. Wainwright, Mandar S. Deshpande, Andrii Iudin, Gaurav Sahni, Jose Salavert Torres, Miriam Hirshberg, Lora Mak, Nurul Nadzirin, David R. Armstrong, Alice R. Clark, Oliver S. Smart, Paul K. Korir, Gerard J. Kleywegt

**Affiliations:** Protein Data Bank in Europe, European Molecular Biology Laboratory, European Bioinformatics Institute (EMBL-EBI), Wellcome Genome Campus, Hinxton, Cambridge, CB10 1SD, UK

## Abstract

The Protein Data Bank in Europe (http://pdbe.org) accepts and annotates depositions of macromolecular structure data in the PDB and EMDB archives and enriches, integrates and disseminates structural information in a variety of ways. The PDBe website has been redesigned based on an analysis of user requirements, and now offers intuitive access to improved and value-added macromolecular structure information. Unique value-added information includes lists of reviews and research articles that cite or mention PDB entries as well as access to figures and legends from full-text open-access publications that describe PDB entries. A powerful new query system not only shows all the PDB entries that match a given query, but also shows the ‘best structures’ for a given macromolecule, ligand complex or sequence family using data-quality information from the wwPDB validation reports. A PDBe RESTful API has been developed to provide unified access to macromolecular structure data available in the PDB and EMDB archives as well as value-added annotations, e.g. regarding structure quality and up-to-date cross-reference information from the SIFTS resource. Taken together, these new developments facilitate unified access to macromolecular structure data in an intuitive way for non-expert users and support expert users in analysing macromolecular structure data.

## INTRODUCTION

The Protein Data Bank in Europe (PDBe; http://pdbe.org; 1) is actively involved in managing three core archives in molecular and cellular structural biology. It is a founding member of the Worldwide Protein Data Bank (wwPDB; http://wwpdb.org; 2), the organisation that manages the Protein Data Bank (PDB), the global archive of 3D atomistic biomacromolecular structures. The wwPDB partners share responsibility for the annotation of macromolecular structure depositions to the PDB with PDBe being responsible for the annotation of all European and African depositions. In the period January to August 2015, the PDBe team annotated over 35% of all the depositions to the PDB. In 2002, PDBe established the Electron Microscopy Data Bank (EMDB; 3) to archive macromolecular structure volumes determined using electron cryo-microscopy (3DEM) and tomography. EMDataBank (emdatabank.org; 4), an international consortium of which PDBe is a founding member, now manages the EMDB. In 2014, PDBe established the Electron Microscopy Pilot Image Archive (EMPIAR; http://pdbe.org/empiar), an archive that stores raw image data for a number of entries in EMDB.

Since the turn of the century, the PDB archive has grown rapidly and now contains more than 110 000 experimentally determined atomic structures. From very early on, with the atomic structure of the DNA double helix (5–7) and the first protein structures of haemoglobin and myoglobin (8,9), to more recently with structures of ribosomes (10,11) and GPCRs (12), structural biology has provided profound insights that aid our understanding of protein structure, evolution, function and their relation to amino-acid sequence. The archiving of atomic structures in the PDB has facilitated the emergence of structural bioinformatics as a field of scientific endeavour and has led to the development of successful methods for the prediction of protein structures (13,14), design of macromolecular inhibitors with potential therapeutic use (15) and design of new protein molecules with desired properties (16).

Unfortunately, due to the complex nature of 3D structural data, users with a limited background or training in structural biology (such as biochemists, molecular biologists, geneticists, medicinal chemists, physicians) do not always find it easy to exploit the rich information content of the structural archives (PDB and EMDB) to help them answer their research questions. It is for archive keepers such as PDBe and the wider structural bioinformatics community to address this challenge and develop new ways of making structural information more easily accessible and relevant to these user communities (17,18). The rapid increase of the typical size and complexity of the molecules and complexes studied poses challenges even for expert users. It is therefore necessary for structural bioinformatics resources to try and understand the evolving requirements of the user communities and to develop innovative methods to address those requirements. In the past few years, PDBe has carried out extensive user studies and identified major challenges faced by experts and non-experts alike:

1. How to obtain accurate, relevant, non-redundant and up-to-date information on macromolecular structure data available in the PDB and EMDB archives?
2. How to assess the quality of models and data, especially when more than one structure is available for a molecule or system of interest?
3. How to understand complex 3D structural data and to use structural information to answer pertinent research questions or to formulate new research hypotheses?

The user study also highlighted an urgent need for innovative methods to make structural data easier to discover and understand:

1. Better methods to query macromolecular structure data, that provide an easy way to identify the best or most relevant structure available in the PDB in a given query context;
2. Better ways to display complex 3D structural data using interactive tools that make it easier for users to understand such data.

Addressing these challenges requires continuous improvements to existing tools and services as well as the development of entirely new tools and data-analysis methods. Over the past decade, PDBe has developed a variety of tools and services to help users find and exploit structural data, e.g. PDBeXplore (19), UniPDB (19), PDBePISA (20), PDBeMotif (21) and PDBeFold (22). In collaboration with the UniProt team, PDBe also maintains SIFTS (23), a resource which links structures in the PDB and sequences archived by UniProt (24) and thereby allows for easy transfer of annotations between the sequence and structure data resources. The information available in SIFTS is central to integrating structural information with other biological data and is used by the wwPDB partners (PDBe, RCSB, 25 and PDBj, 26) and many other structural bioinformatics resources (e.g. CATH, 27, SCOP, 28, Pfam, 29, InterPro, 30, Reactome, 31) as well as a variety of academic research teams. The additional annotations available from SIFTS are now integrated in visualisation tools such as JSmol (http://www.jmol.org/) and JalView (32). These and other visualisation tools such as OpenAstexViewer (33), PyMOL (www.pymol.org) and Chimera (34), and interactive web-based interfaces (e.g. Vivaldi, 35) are important for making analysis and visualisation of 3D structural data more accessible for non-expert and expert users alike.

These efforts, alongside the archive-remediation work by the wwPDB partners to improve the quality and consistency of the data in the PDB archive (36), have helped PDBe to improve the data-discovery mechanism. Additionally, query systems have been enriched by incorporating value-added annotation from SIFTS and other chemical and biomedical resources such as ChEMBL (37) and DrugBank (38), and genomic information such as gene names and homologous protein information from the Homologene resource (http://www.ncbi.nlm.nih.gov/homologene). All these improvements help to address the problem of finding information in the PDB that is relevant to many types of queries by reducing the false-positive rate. For all search systems to date, the fundamental unit of information remains a PDB entry rather than the specific biological molecule or assembly studied in a given experiment. As a consequence, with the growth of the PDB beyond 110 000 structures, the result sets may include long lists of PDB entries, and their use and analysis becomes time-consuming, as users may have to manually sort through them. To address this issue, it is necessary to radically improve the ways in which macromolecular structure data can be queried and to provide mechanisms for basic analysis of result sets, e.g. by identifying a representative (or ‘best’) structure for every molecule or complex (instead of a long list of duplicates, variants, mutants and complexes), by making it easy to narrow down search results, by providing additional ways of displaying results other than long lists of entries and by displaying key summary information for each entry in an intuitive manner.

Here we describe the results of recent efforts at PDBe to improve the accessibility of macromolecular structure data (‘redesign project’) by:

- Improving the quality of the metadata of entries in the PDB archive;
- Adding annotations and value-added information to provide a biological context for all structural data in the PDB;
- Enhancing data accessibility by developing a RESTful API to provide unified access to all macromolecular structure data;
- Addressing issues related to the data-query mechanism and basic analysis of search results;
- Redesigning the web pages based on user-centric design principles and understanding of user requirements.

## IMPROVING METADATA QUALITY

Since 2003, the wwPDB partners have carried out a number of extensive archive-remediation projects to advance the quality, consistency and integrity of the information present in the PDB (36), and they continue to improve the archive, for instance through enhancing the representation of small molecule data (especially peptidic antibiotics and inhibitors, 39). These efforts have resulted in better data quality and have also led to improved annotation practices. Such remediation, however, is an on-going and labour-intensive process. In the redesign project described here, PDBe has addressed several additional data-consistency issues (Table 1) and a number of user requests such as making information on intramolecular connectivity available as part of the entry description and having consistent representation of ligand-binding data. These enhanced data are loaded into PDBe's Oracle database that powers the PDBe services and web pages. Enhancing the data quality in the archive PDBx/mmCIF files and making that information available via the central database ensures that all PDBe services provide users with improved and consistent information.

**Table 1.** A list of PDBx/mmCIF data items that are made consistent in all entries in the PDB archive before the data are loaded into the PDBe database. The second column summarises the changes made to each data item to make it consistent

_struct_ref_seq_dif.details	Describes the observed differences between a residue in the PDB and corresponding residue in reference database (e.g. UniProt for protein molecules)
_exptl.method	Describes the experimental methods used to determine the 3D structure.
_diffrn_source.source	The radiation source used to carry out the diffraction experiment, e.g. ‘Rotating anode’
_citation.journal_abbrev	The abbreviated name of the cited journal
_diffrn_radiation.pdbx_diffrn_protocol	Diffraction protocol used, e.g. ‘Single wavelength’, ‘MAD’ etc.
_diffrn_detector.detector	The type of detector used in the diffraction experiment
_computing.structure_refinement	Software used for refinement of the structure
_symmetry.space_group_name_H-M	Space group symmetry

## VALUE-ADDED INFORMATION

Integrating PDB data with information from other biological data resources has long been a priority for PDBe. More than a decade ago, these efforts resulted in the SIFTS resource which continues to provide up-to-date cross-reference information between PDB and UniProt. This information has made it possible to develop an automated procedure to obtain consistent names for the protein molecules across the PDB archive, based on the recommended names, other names and feature-table information available in UniProt. Only if there is no UniProt cross-reference available are the macromolecule names from the PDB entry used. SIFTS information has also made it possible to provide information on gene names and homologous proteins, derived from UniProt and Homologene.

PDB annotation includes the ‘quaternary structure’ or the predicted assembly of the macromolecules in the crystal. The assemblies are predicted using PISA (20), which was developed jointly by PDBe and CCP4 (40). When predicting possible assemblies, PISA calculates additional information including the accessible and buried surface areas, interacting residues in the interface(s) of multimeric assemblies and binding energies. These types of information are very valuable when studying protein–protein interactions. As part of the redesign project, every assembly annotated in the PDBx/mmCIF file of a PDB entry is explicitly generated and the PISA information for it is stored. A succinct textual description of the assembly composition is also derived automatically, e.g. ‘protein structure’ for monomers and homomeric oligomers; ‘protein–protein complex’ for heteromeric complexes containing only proteins; ‘DNA–Protein complex’ or ‘RNA–Protein complex’, etc.

The recent adoption of PDBx/mmCIF as the principal distribution format for the PDB archive has brought about many improvements in the way in which macromolecular structures can be represented. For instance, with PDBx/mmCIF it is possible to represent large structures, such as complete ribosomes, in a single file. The file format is extensible and thus allows for local extensions enriching the information available in these files. At present, the PDBx/mmCIF files in the PDB archive do not contain any connectivity and bond-order information for any of the standard or non-standard residues and bound molecules. This poses a challenge for molecular graphics software (and other software that needs to ‘perceive’ the chemistry of entities), as these usually only store the connectivity of standard residues. As part of the new developments at PDBe, the information describing non-standard residues, small molecules and their binding sites has been improved across the archive, by adding connectivity and binding-site information in a consistent way for all non-standard small molecules present in the PDB entry. This information is then made available to all PDBe services through its database. We have further modified the OpenAstexViewer to read the connectivity information directly from PDBx/mmCIF files to allow for better graphic presentation of small molecules. The information is also used to produce static images for the PDBe entry pages (using PyMOL version 1.6) that accurately portray the small molecule connectivity. The updated PDBx/mmCIF files are available through the PDBe entry pages.

**Figure 1.**
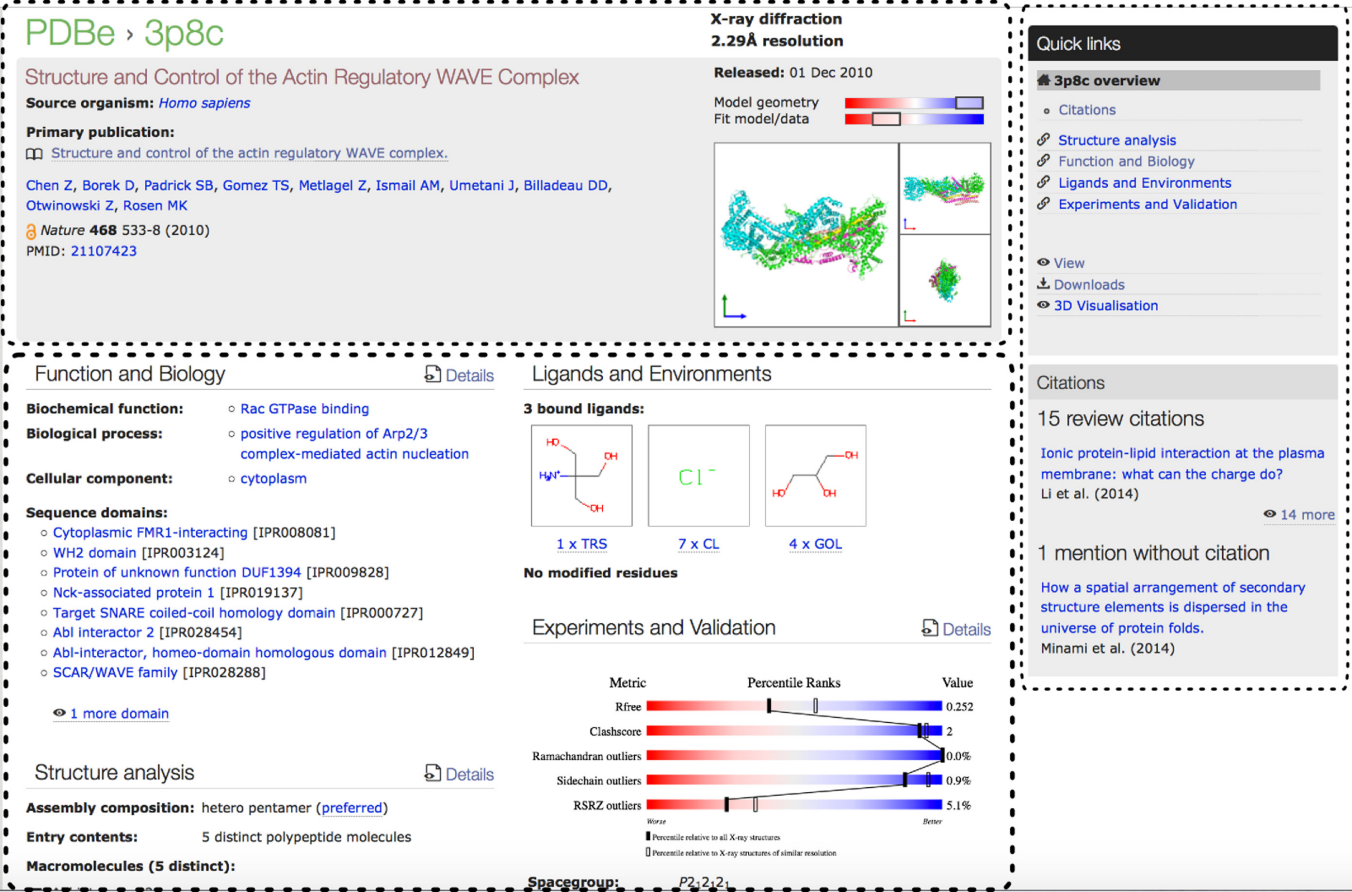
An example of an entry summary page showing the organisation into three main areas (highlighted with dashed lines). The top panel shows the essential details and access to a picture gallery. The right-hand panel contains ‘Quick links’ to pages with more detailed information, file downloads and 3D viewer. It is also used to provide other relevant information, e.g. if the entry has been cited or mentioned in reviews or other articles. The main body of the page is divided into four sections as described in the text, providing summary information and links to the detailed pages for each of the sections.

As part of the wwPDB collaboration, PDBe has implemented a validation pipeline (41) based on the recommendations of the X-ray Validation Task Force (VTF) (42). The pipeline has been in production at all wwPDB sites since August 2013 and a validation report for every PDB entry solved by X-ray crystallography is made available in PDF format via the wwPDB FTP sites. Additionally, detailed validation information is made available in XML format via the same sites. The XML data are loaded into the PDBe database and thus available to all PDBe services and web pages. One of the major recommendations of the wwPDB X-ray VTF was to produce percentile plots for a number of key validation-related parameters for every entry (42). While these plots are displayed on the new PDBe entry pages, a more compact representation is also needed that provides a uniform, condensed, at-a-glance impression of the quality of structures. Such a combined quality measure can be calculated by aggregating quality information regarding the model on the one hand and the fit of the model to the experimental data on the other. In practice, the combined model-quality measure is the harmonic average of the percentile scores for the applicable model-quality indicators (such as clash-score (43), percentage of residues classified as Ramachandran outliers and rotamer outliers), whereas the agreement between model and data is represented by the harmonic average of the *R*_free_ and RSR-Z outlier percentile scores. (If no experimental data were deposited, the overall quality score is reduced by half.) The harmonic averages are graphically ‘blurred’ so as not to suggest too high a degree of numerical precision/accuracy (it is unlikely that the quality of a structure with a harmonic average score of 85% is significantly different from one with a value of 80% or 90%, so such scores are made visually indistinguishable). The resulting representation is compact enough to be shown by many PDBe services to highlight the quality of individual PDB entries (Figure 1). Finally, a single quality indicator is also calculated for each entry by taking the harmonic average of all the percentile scores representing model and model-data-fit quality measures and then subtracting 10 times the numerical value of the resolution (in Ångström) of the entry to ensure that resolution plays a role in characterising the quality of a structure. This single empirical ‘quality measure’ value is used by the PDBe query system to sort results and identify the ‘best’ structure in a given context. At present, entries determined by methods other than X-ray crystallography do not have similar data quality information available and are not considered as ‘best structures’.

Usually, the only way to obtain information about the goals of structural studies and the biological insights gained from them is to access the publication alongside the deposited data available in the various archives, since relatively little biological information about the biological goals and results of a structure determination is captured as part of PDB deposition or annotation. Accessing relevant publications that cite or mention a given PDB entry can further contribute to the understanding of the biological relevance of a given structure. Locating the publication in Europe PMC (http://europepmc.org/) or PubMed (http://www.ncbi.nlm.nih.gov/pubmed) allows users to identify all relevant publications that cite it, but it is much more difficult to find publications that mention a PDB code but do not reference the paper in which it was described. Moreover, about one in six PDB entries are never published at all and hitherto identifying publications that cite such structures has been almost impossible. To address these problems, PDBe has collaborated with the Europe PMC team at EMBL-EBI to identify through text mining (of full-text, open access) publications that mention PDB codes but do not refer to any publication describing the PDB entry. Moreover, through Europe PMC, PDBe now has access to all the figures and figure legends for full-text open-access publications describing PDB entries.

The value-added information described in this section is loaded into the PDBe database and the next section describes various ways in which it is made accessible to PDBe users, tools and services.

## IMPROVING DATA DISSEMINATION

With improved data quality and value-added information now available, PDBe has developed ways to make these accessible with a focus on two major communities:

1. User communities that require programmatic access to structural and structure-related information (e.g. bioinformaticians and chemoinformaticians).
2. Non-experts, experts and occasional users of the PDB who require access to this information via the web and who may only be interested in a single entry.

To support these two distinct use cases we have developed a RESTful API and new web-based entry pages as well as a powerful new query system, which can be accessed via the RESTful API or a web interface.

## PDBe RESTful APPLICATION PROGRAMMING INTERFACE (API)

The RESTful API (http://pdbe.org/api) was designed to provide programmatic access to all information in the PDBe database. This includes information available in the PDB and EMDB archives, but also the improved and value-added information described above. The API also makes information from other PDBe tools and services (such as PISA) programmatically available. For easy access, the API contains separate modules for PDB, EMDB, SIFTS, PISA and validation information with relevant calls aggregated in each module. This allows for easy integration of all macromolecular structure information into an application or workflow. The API also makes it possible to access structure-related information in manageable data blocks without having to read large files or parse data that is not relevant.

The data sources underlying the API are updated weekly and the API is tested extensively with every week's release of new data into the archives. Importantly, the API is integrated in the PDBe production workflow and supports the redesigned PDB and EMDB entry pages. This ensures that the API is reliable, stable and provides up-to-date information. The API has been integrated in Jmol/JSmol where it is used to show value-added annotations from SIFTS as well as validation-related information. JalView has also integrated the API to provide its users with a facility to query PDB data and to provide additional value-added information for individual PDB entries. We envision the PDBe API to be a long-term, stable, well-tested resource that powers traditional and novel applications based on 3D structural information.

## ENTRY PAGES

The PDBe entry pages that provide information for every PDB and EMDB entry have been completely redesigned following an extensive user survey, user testing and feedback to facilitate intuitive and easy data accessibility. PDB and EMDB entry pages now have the same organisation and layout, making it easier for users to access data in both archives. By following best practices for ‘responsive design’, we have also ensured that the pages and all the newly developed tools have a user-friendly interface on different devices such as desktops, tablets and smartphones.

The summary page for an entry is organised in three main areas as shown in Figure 1. The top of the page provides succinct information about structure quality, experimental method(s), entry title and the publication that describes the structure. It also provides access to a picture gallery containing a variety of images of the deposited entry and the macromolecular assembly. The right-hand side of an entry page contains ‘Quick links’ to pages with more detailed information, file downloads, etc. as well as other information that is relevant in the context of the entry and the page. For example, on a summary page of a PDB entry there are pointers to reviews that cite that PDB entry as well as to papers that mention the PDB entry but do not cite the original publication.

The remainder of the page is divided into four panels that provide summary information about important categories of information regarding the structure and provide links that enable the user to ‘drill down’ to obtain more details. The content of the four panels is based on analysis of a study we carried out with users from different backgrounds and specialisations, including biochemists, structural biologists, clinicians, geneticists and others, from both academia and industry. These users were provided with several dozen cards that each contained the name of a concept or information item that is available for (many) PDB entries, e.g. ‘structure quality’, ‘assembly’, ‘biological function’, ‘sequence domain’. The users were asked to link concepts that they considered to be related. The resulting links were subjected to cluster analysis and clearly revealed four major clusters of concepts that describe how users think about structures: ‘Function and Biology’, ‘Ligands and Environments’, ‘Structure analysis’ and ‘Experiment and Validation’. Each of these four categories is represented in a separate panel, and additional information for each can be obtained through one or more levels of detail pages. An additional detail page brings together information on the literature related to the entry.

Thus, there are five types of entry-detail pages that contain the following information for a given entry:

1. Citations—The citation page shows the publication that describes the PDB entry. It also shows the figures and legends from the primary citation if the publication is ‘full-text open access’. In addition, the page lists reviews and articles that cite or mention the PDB code of the entry, thus helping users to discover relevant publications.
2. Structure analysis—The structure analysis page lists all the assemblies (quaternary structures) annotated for the PDB entry. The PDB assemblies have been analysed by PISA and, where available, a summary of information on each assembly is listed (such as accessible and buried surface area and calculated dissociation energy). This page also lists each unique macromolecule and associated cross-reference information to structure and sequence family databases such as gene names, UniProt accessions, etc. The page contains an interactive sequence-feature viewer that shows annotations mapped onto the sequence of each individual molecule. Additionally, there are links to detail pages that describe each individual macromolecule and contain interactive viewers to help users understand their 3D structure.
3. Function and Biology—This page addresses the biological relevance of the structure. It includes information about the biological process, function and cellular location based on the Gene Ontology (GO) (44) assignments provided by SIFTS. For enzymes, information is shown on the reaction catalysed and other relevant comments on function or biological availability of the particular enzyme obtained from enzyme resources such as IntEnz (45), Brenda (46) and Expasy (47). The page also contains information on sequence (Pfam; 29) and structure domains (CATH; 27 and SCOP; 28) and has a gallery of images that depict the assemblies and the mapping of the sequence and structure domains onto the 3D structure.
4. Ligands and Environments—For each unique bound molecule, there is a page with general information about the molecule, and a 2D and optional 3D view of the environment where each instance of the molecule is bound. The 2D representation of the binding site uses the program LigPlot (48) from PDBsum (49).
5. Experiment and Validation—This page lists experiment-related information provided by the depositor and a summary of data that can help users assess the quality of the structure. The quality information is taken from the corresponding wwPDB validation report (42).

Images of the PDB entry or the assembly are a very effective way not only to provide a simple view of the molecule but also to convey information and provide additional insights into the structure, e.g.

- Identifying a single macromolecule in the assembly;
- Identifying the number of unique macromolecules that are present in a complex;
- Showing the overall shape of a complex;
- Depicting annotation such as sequence or structure domains on the macromolecular structure.

**Figure 2.**
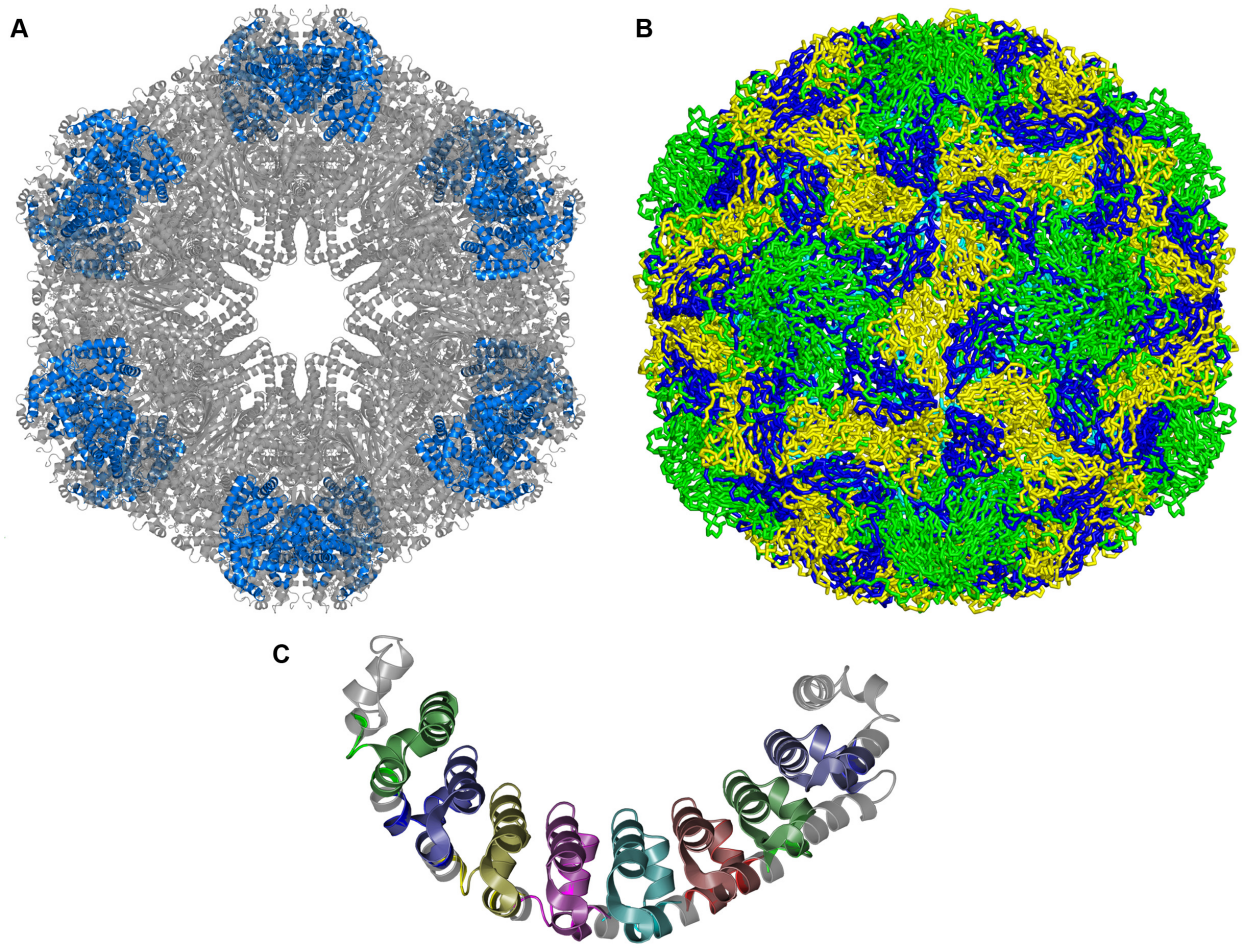
(**A**) Image highlighting the location of the six copies of globin d (blue) within the giant haemoglobin complex from *Glossoscolex paulistus*. PDB entry 4u8u. (**B**) The human rhinovirus capsid (PDB entry 4rhv) contains 60 copies each of four different proteins: VP1 (green), VP2 (yellow), VP3 (blue) and VP4 (cyan). (**C**) The location of the eight copies of Pfam domain ‘Pumilio-family RNA binding repeat’ is highlighted in this image of Human Pumilio 1 protein, PDB entry 3bsx.

To facilitate such insights, visualisation programs need to understand what the unique small molecules and macromolecules are in a PDB entry so that they can be highlighted individually. PDBe has worked closely with the PyMOL developers at Schrödinger to implement additional functionality in PyMOL. Figure 2 shows examples of images coloured by unique molecules or annotations that can help users to understand complex structure data.

**Figure 3.**
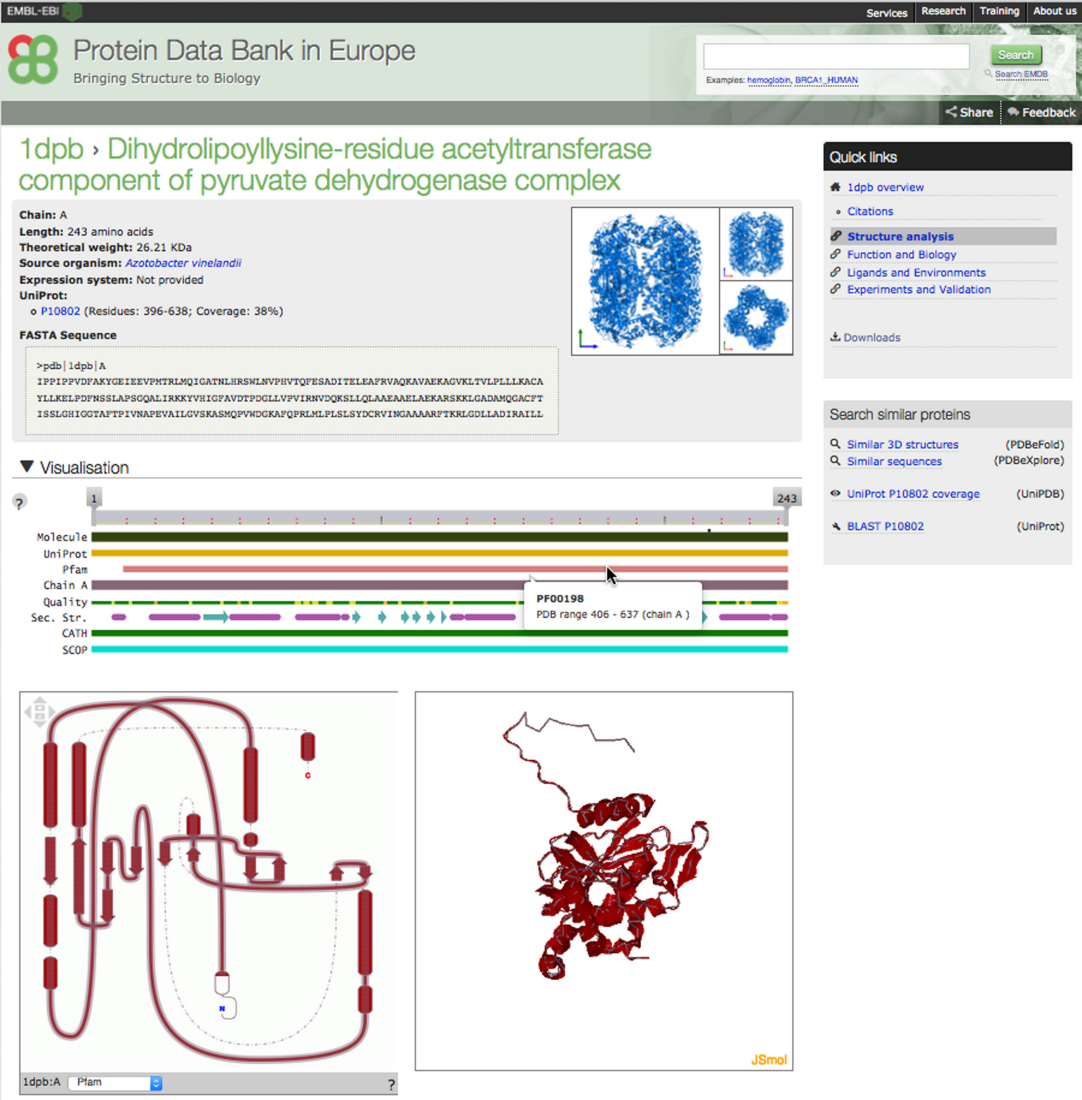
Interactive visualisation tools displaying annotations for each individual protein molecule. The figure shows the Pfam domain (PF00198: 2-oxoacid dehydrogenases acyltransferase (catalytic domain)) in protein molecule 'Dihydrolipoyllysine-residue acetyltransferase component of pyruvate dehydrogenase complex' (PDB entry 1dpb) highlighted in the sequence feature view (1D), topology diagram (2D) and JSmol (3D) viewer that are interlinked to show annotations in 1D, 2D and 3D.

A number of interactive tools have been incorporated into the PDBe web pages to help users analyse macromolecules and ligands in PDB entries. An example of a page that describes each unique macromolecule in a given entry is shown in Figure 3. This page, linked from both the ‘Structure analysis’ and ‘Summary’ pages (as ‘Molecule details’), provides summary information, including the name of the macromolecule, its sequence in FASTA format, gene name, source organism and expression system. It also contains a gallery of pictures highlighting the given macromolecule in the ‘preferred’ assembly or complex as well as highlighting all the sequence (Pfam) and structure domains (SCOP and CATH). The page contains three interactive viewers that present information on 1D (sequence-feature viewer), 2D (topology viewer) and 3D structure (using JSmol; Figure 3). For each chain, the sequence-feature view shows the sample sequence studied and depicts value-added annotation from SIFTS including residue-level mapping to UniProt, sequence families (Pfam), structure domains (SCOP, CATH), binding-site residues, structure quality and the secondary structure. By default the sequence-feature view shows the chain that has the maximum number of observed residues. The same chain is also shown in the topology viewer, which depicts the helices and strands in a 2D representation that takes into account the interactions of these secondary structure elements, leading to a consistent display of sheets and domains in the structure. JSmol is used for interactive display of the 3D structure. The three viewers are linked and selecting a feature in the sequence or topology view shows the same in the other two views including the 3D structure view in JSmol. The interactive topology view helps users link (and understand) sequence-based annotation to the 3D molecular structure view.

## IMPROVED QUERY MECHANISM

There are many query systems available for PDB data, both at wwPDB partner sites and at other structural bioinformatics resources such as PDBsum and OCA (http://oca.weizmann.ac.il). These query systems usually include information annotated in the PDB entries and value-added information (e.g. from SIFTS) or cross-references and value-added information from other resources such as ChEMBL, UniProt and Drugbank or genome information from Ensembl (50). Each query system has its unique features, strengths and weaknesses, but they all typically present the results of a query as a list of PDB entries. This is useful for an expert user who can deal with a large number of PDB entries, but for a non-expert it can be confusing when a number of PDB entries are listed for essentially the same protein (e.g. Human carbonic anhydrase 2 features in 541 entries as of 17 September 2015). The problem is compounded by the fact that it is not easy to assess and compare the quality of such sets of related structures. One way to alleviate this problem is to have ‘facets’, i.e. a facility that allows users to drill down to a single or a manageable number of PDB entries based on other filtering criteria such as ‘organism’, ‘sequence or structure domain’, ‘resolution’, etc. Many query systems also offer an ‘autocomplete’ feature that helps narrow down the search by providing useful matching terms as users type their query. This usually reduces results sets to a more manageable number of PDB entries with fewer false positives, but users still have to assess all entries to select the one(s) most suitable for their purposes. Moreover, such features still do not support queries such as:

1. Find all the structures for a given class of macromolecules (e.g. kinases) in the PDB and identify the best structure for each unique macromolecule;
2. Identify all proteins and small molecules in the PDB which have been observed to interact with a given protein;
3. List all organisms from which structures for a particular protein are available;
4. Generate a list of all unique proteins that contain a given Pfam domain and have structures in the PDB, with the best representative PDB entry for each unique Pfam domain and protein combination;
5. Identify the best structure, based on validation information, for a given protein amongst many available in the PDB.

**Figure 4.**
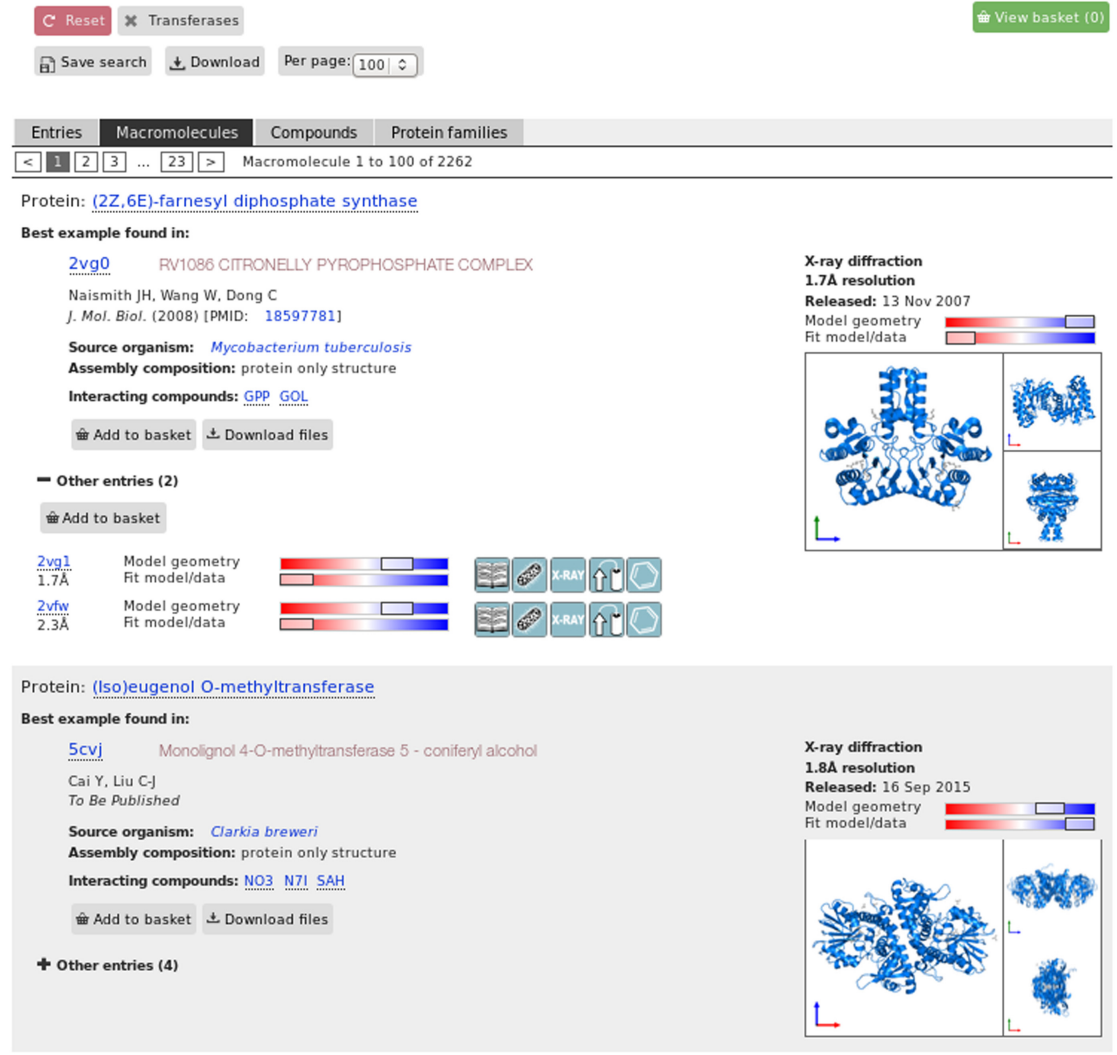
The ‘macromolecule’ view of the query interface. The results show the number of unique macromolecules (in this case 2262) present in the result set for the query ‘transferases’. A representative (or ‘best’) structure for each unique macromolecule is shown, with an image gallery highlighting the location of the particular macromolecule within the assembly. Other instances of this macromolecule in PDB entries are listed below. This is in addition to the traditional result interface which lists all the PDB entries that satisfy the given query criterion (≈18000 individual PDB entries in this case).

The new PDBe query system contains several features available in existing systems (such as providing facets and autocomplete), but also allows for some basic analysis of result sets (such as identifying the best structure or the number of unique proteins in a set) and thus supports the types of query listed above. This is accomplished by offering different ways to present the results, e.g. as a simple list of PDB entries (‘Entries view’), or as a list of unique macromolecules found in the result set, and by identifying the best PDB entry (based on the combined quality criterion described earlier) for each unique macromolecule (‘Macromolecules view’), a list of the best available PDB entries for each macromolecule that binds to a given small molecule in the result set (‘Compounds view’), or a list of the best PDB entries for each protein family in the result set (‘Protein families’ view). Figure 4 shows a ‘Macromolecules view’ for the results of the query ‘transferases’ selected from the enzyme category in the autocomplete suggestions, listing the best structure for each of the 2262 unique molecules in the PDB archive (as of 17 September 2015) that are classified as transferase enzymes.

The query system uses the Apache-Solr indexing engine and the additional functionality that provides ‘refine query’ functionality was developed as part of the ‘BioSolr’ project. The results interface was developed using an updated version of Ajax-Solr (https://github.com/evolvingweb/ajax-solr), a JavaScript framework for creating user interfaces to Solr.

## PDBe WEBSITE

The PDBe home page (http://pdbe.org) has also undergone re-organisation to reflect the new developments and to consolidate the available functionality. The ‘PDBe services’ tab provides direct access to the advanced PDBe tools, services and resources and also categorises these based on a user's main area of interest. Each category lists the most relevant tools and services and provides a short explanation of the functionality available. Efforts have been made to consolidate all the PDBe training material and related resources under the ‘PDBe training’ tab. The resources are categorised to provide easy access to teaching materials and tutorials. PDBe presentations and webinars are also made available.

## FUTURE DEVELOPMENTS

The work to improve the data accessibility will continue with the next stage focused on information related to small molecules, binding sites, integration of data quality information for all experiment types and enhancements to the value-added annotation by including genomic information including variation and SNP data. PDBe has several services that provide information on small molecules (PDBeChem, 51) and their binding sites (PDBeMotif, 21). These services will be refactored and integrated into the new infrastructure to make the information more accessible. PDBe will continue to work on improving the user interfaces and query mechanisms and making additional data available via the REST API to meet the needs of expert and non-expert users alike.
